# β-Carotene from Yeasts Enhances Laccase Production of *Pleurotus eryngii* var. *ferulae* in Co-culture

**DOI:** 10.3389/fmicb.2017.01101

**Published:** 2017-06-16

**Authors:** Chaolin Guo, Liting Zhao, Feng Wang, Jian Lu, Zhongyang Ding, Guiyang Shi

**Affiliations:** ^1^Key Laboratory of Carbohydrate Chemistry and Biotechnology, Ministry of Education, School of Biotechnology, Jiangnan UniversityWuxi, China; ^2^National Engineering Laboratory for Cereal Fermentation Technology, Jiangnan UniversityWuxi, China; ^3^School of Food and Biological Engineering, Jiangsu UniversityZhenjiang, China

**Keywords:** co-culture, laccase, β-carotene, *Pleurotus eryngii* var. *ferulae*, yeast

## Abstract

Laccase is widely used in several industrial applications and co-culture is a common method for enhancing laccase production in submerged fermentation. In this study, the co-culture of four yeasts with *Pleurotus eryngii* var. *ferulae* was found to enhance laccase production. An analysis of sterilization temperatures and extraction conditions revealed that the stimulatory compound in yeasts was temperature-sensitive, and that it was fat-soluble. An LC-MS analysis revealed that the possible stimulatory compound for laccase production in the four yeast extracts was β-carotene. Moreover, the addition of 4 mg β-carotene to 150 mL of *P. eryngii* var. *ferulae* culture broth improved laccase production by 2.2-fold compared with the control (i.e., a monoculture), and was similar to laccase production in co-culture. In addition, the enhanced laccase production was accompanied by an increase of *lac* gene transcription, which was 6.2-time higher than the control on the fifth day. Therefore, it was concluded that β-carotene from the co-cultured yeasts enhanced laccase production in *P. eryngii* var. *ferulae*, and strains that produce β-carotene could be selected to enhance fungal laccase production in a co-culture. Alternatively, β-carotene or crude extracts of β-carotene could be used to induce high laccase production in large scale.

## Introduction

Laccase (EC 1.10.3.2) is a group of blue multi-copper oxidases that are widely distributed in microorganisms, plants and insects, and capable of catalyzing the reaction various organic compounds with the reduction of oxygen. Due to its excellent activity, laccase is widely used in several applications, including dye treatment in the textile industry (Shraddha et al., 2011), pulp bleaching in the paper industry (Jeon and Chang, 2013), and in the food industry (Brijwani et al., 2010). Laccase is mainly produced by fungi, and co-culture is an effective method to optimize laccase production in submerged fermentation (Wang et al., 2009; Flores et al., 2010; Li et al., 2011).

Due to its wide application, many researchers have focused on increasing laccase production in submerged fermentation. Yeasts are hosts that are well-known to express various recombinant proteins. Therefore, some laccase-encoding genes have been isolated from fungi, and recombinant laccase production was effectively enhanced in yeasts (Klonowska et al., 2005; Hong et al., 2007). Besides genetic engineering technology, enhanced laccase production has also been achieved by optimizing the culture medium (Dekker et al., 2007), especially by adding inducers. Some researchers have focused on screening for effective inducers, and laccase production in *Trametes versicolor* has been effectively improved by farnesol and chitosan (Adekunle et al., 2015; Hu et al., 2016). Different inducers enhance laccase production by various mechanisms, including the improvement of silent or poorly expressed laccase isoforms and the response to oxidative stress caused by an inducer (Hoegger et al., 2006; Wang et al., 2014). The elucidation of these mechanisms has been beneficial to the screening of more inducers that enhance laccase production.

Currently, laccase production in white rot fungi (WRF) has been effectively enhanced with other microbial strains, including yeasts and fungi, in a co-culture fermentation (Dwivedi et al., 2011; Dong et al., 2012). Some studies have focused on the determining the mechanism of the enhancement of laccase production in a co-culture fermentation with different fungi. Yeast growth led to glucose starvation in a co-culture, which caused the overexpression of *T. versicolor* laccase (Wang et al., 2009). In addition, laccase production by *Ganoderma lucidum* may be enhanced by glycerol as a second carbon source, which was produced from glucose by the yeast in a co-culture (Li et al., 2011). Moreover, enhanced laccase production in fungi may be caused by some compounds in the culture broth of the co-cultured microbial strains (Crowe and Olsson, 2001). However, these reports did not indicate a common mechanism, and no specific compound was found to stimulate laccase production in co-culture. The discovery of unequivocal stimulatory compounds will be helpful to screen specific strains for co-culturing with fungi to improve laccase production.

In our previous study, laccase production of *Pleurotus eryngii* var. *ferulae* (Lanzi) Sacc. was significantly enhanced in a co-culture fermentation with *Rhodotorula mucilaginosa* (Wang et al., 2015). To determine the reason, previously reported possible mechanisms were all investigated, but none were responsible for enhanced laccase production by *P. eryngii* var. *ferulae* in a co-culture. In the present study, we found that the putative stimulatory compound in the co-cultured yeast was sensitive to the sterilization temperature. We then identified the stimulatory compound in the co-culture as β-carotene, and the mechanism of stimulation was also investigated.

## Materials and Methods

### Strains and Culture Conditions

The laccase-producing fungus used in this study, *Pleurotus eryngii* var. *ferulae* (Lanzi) Sacc. JM301, was maintained in our laboratory on potato dextrose agar (PDA) slants at 4°C. Four yeast strains, *R. mucilaginosa, Phaffia rhodozyma, Sporidiobolus pararoseus* and *R. glutinis*, were also maintained in our laboratory, and used in co-culture with the laccase-producing fungi. The culture medium for *P. eryngii* var. *ferulae* was composed of: 20 g/L glucose, 20 g/L corn powder, 20 g/L wheat bran and 0.1742 g/L K_2_SO_4_ (pH 9.0). Fungal mycelia were inoculated in the culture medium and incubated at 30°C and 150 rpm. The culture medium for the yeast was YPD (10 g/L yeast extract, 20 g/L peptone and 20 g/L glucose) and cells were incubated at 30°C and 200 rpm.

### Co-culture of Laccase-Producing Fungi with Yeasts

Inoculations were done as in our previous study, with slight modifications (Wang et al., 2015). The yeasts used in this study were cultured under the conditions described above for 48 h, and then inoculated into a fungal culture that had been cultured for 2-days. The inoculum size of the yeast was 3% (v/v) and the culture time was 5 days after inoculation. To investigate the effect of the sterilization temperature, *R. mucilaginosa* cells cultured for 48 h were exposed to different temperatures (70, 80, 90, 100, 115, and 121°C) for 1 h, and then 3% (v/v) was inoculated into the monoculture of *P. eryngii* var. *ferulae* as above condition.

### Protease Treatment of *R. mucilaginosa*

In this study, four proteases were used to treat sterilized *R. mucilaginosa* cells at 70°C. Four milligram of centrifuged cells were dissolved in 4 mL of distilled water and treated with four different commercial proteases, including 1 mg/mL snailase (Sangon Co., Shanghai, China) and 0.2 mg/mL pepsin (Sigma Co.), trypsase (Sangon Co.) and proteinase K (Thermal Fisher Scientific Co.). The protease treatment was carried out at 37°C for 5 h. The treated cells were washed with sterilized water and collected by centrifugation twice. The centrifuged protease-treated cells or 4 g of untreated cells were dissolved in 4 mL of sterilized water and inoculated into the fungal culture broth on day 2.

### Extraction of Yeast Cells

*Rhodotorula mucilaginosa* and other yeast cells were collected by centrifugation and 4 g of cells were dissolved in 4 mL of distilled water. The cells were treated eight times repetitively with freezing and thawing, then extracted with 70°C distilled water for 1 h and centrifuged at 7104 × *g* for 10 min. The supernatant was designated as the W-extract of the yeast cells. Precipitates were extracted with 5 mL of trichloromethane at room temperature for 1 h, and the solutions were concentrated by rotary evaporation. The extracts were dissolved in 1 mL of plant oil and the supernatant was designated as the O-extract of the yeast cells. The W- and O-extracts of the yeast cells were added to *P. eryngii* var. *ferulae* under the co-culture conditions described above and the laccase activity was determined. The solvents used for the extraction were aseptically filtered.

### Addition of β-Carotene

To identify the putative stimulatory compound, 1–4 mg of β-carotene of purity ≥96.0% (Klamar, Shanghai, China) was dissolved in plant oil and the solution was sterilized by aseptic filtration. Different amounts of β-carotene were added to 150-mL of *P. eryngii* var. *ferulae* culture medium after 48 h of culture. The mycelia of *P. eryngii* var. *ferulae* were cultured for 5 days after the β-carotene was added, and the laccase activity in the culture broth was determined each day.

### LC-MS

The four yeasts, *R. mucilaginosa, Phaffia rhodozyma, S. pararoseus* and *R. glutinis*, were co-cultured with *P. eryngii* var. *ferulae*. The yeast cells were collected by centrifugation and then ground in liquid nitrogen. The ground yeast cells were extracted with water and trichloromethane as mentioned above. The similar compounds in the different yeast co-cultures were separated and identified with an Ultra Performance LC system, which was connected to a Waters MALDI Q-TOF mass spectrometer (MS). Extracts of different yeasts were separated in a reverse-phase C18 column (Waters, Milford, MA, United States), and eluted with mobile phases of acetonitrile–isopropanol (10:90, v/v) (A) and acetonitrile (B) at flow rate of 0.3 mL/min in a gradient program (Supplementary Table 1). The separated compounds were then detected with a MS (Waters, Milford, MA, United States). Five carotenoid standards, zeaxanthin, lycopene, astaxanthin, xanthin and β-carotene, were also assayed by the same method.

### Quantitative Real-Time PCR (qRT-PCR)

*Pleurotus eryngii var. ferulae* mycelia cultured with β-carotene were collected by centrifugation and ground after freezing in liquid nitrogen. The mycelia were suspended in phosphate buffer (pH 7.0), and the total RNA was extracted with a Trizol Total RNA Purification Kit (Sangon; Shanghai, China). Reverse transcription was carried out using a RevertAid First Strand cDNA Synthesis Kit (Fermentas; Burlington, ON, Canada) to obtain cDNA. The transcription level of *lac* was determined by qRT-PCR using SYBR green (TaKaRa, Japan). The primers used in this study are listed in Supplementary Table 2. The gene expression levels were normalized against the level of *rns*, as reported previously (Li et al., 2013).

### Biochemical Analysis

The laccase activity was determined according to a method reported previously (Hou et al., 2004). The 1-mL reaction mixture contained 880 μL of 100 mM sodium acetate buffer (pH 4.5), ABTS solution at a final concentration of 1 mM and 20 μL of diluted enzyme solution. The laccase activity was calculated using the molar extinction coefficient of oxidized ABTS (𝜀420 = 3.6 × 10^4^ M^-1^ cm^-1^). A unit of laccase was defined as the amount of enzyme required to oxidize 1 μmol of ABTS per minute.

### Statistical Analysis

All experiments were performed in triplicate and the data were expressed as the mean ± SD. Significant differences (*p* < 0.05) between means were analyzed using GraphPad Prism 5.0 software.

## Results

### Laccase Production of *P. eryngii* var. *ferulae* Co-cultured with Yeast

**FIGURE 1:**
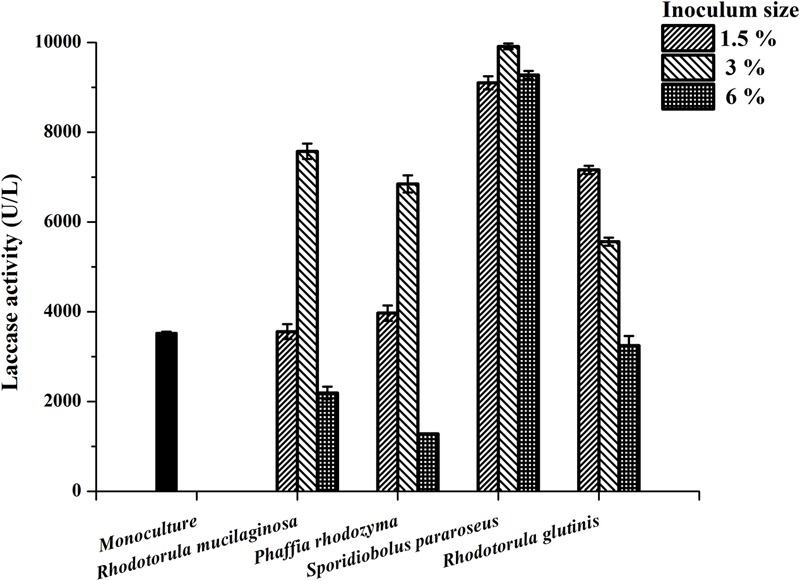
Laccase production in co-cultures of *P. eryngii* var. *ferulae* and four yeasts.

Our previous study indicated that the yeast *R. mucilaginosa* improved the laccase production of *P. eryngii* var. *ferulae* JM301 in co-culture (Wang et al., 2015). In the present study, several yeasts were co-cultured with *P. eryngii* var. *ferulae* JM301 and the laccase production was determined. The results showed that three of the yeasts, *Phaffia rhodozyma, S. pararoseus* and *R. glutinis*, significantly enhanced laccase production in co-culture, and the highest laccase production (9911 U/L) by *P. eryngii* var. *ferulae* was obtained from a co-culture with *S. pararoseus* inoculated at 3% (**Figure 1**). The three yeasts all had a stimulatory effect on the laccase production, which was similar to that seen with *R. mucilaginosa*. As the inoculum size was increased from 1.5 to 6%, the laccase production in co-culture peaked at 3%, and was reduced at 6%. Differently than for the other three yeasts, laccase production in a co-culture of *P. eryngii* var. *ferulae* and *R. glutinis* decreased as the inoculum size varied from 1.5 to 6%.

### Effects of Extraction Conditions of Yeasts on Laccase Production in Co-culture

**FIGURE 2:**
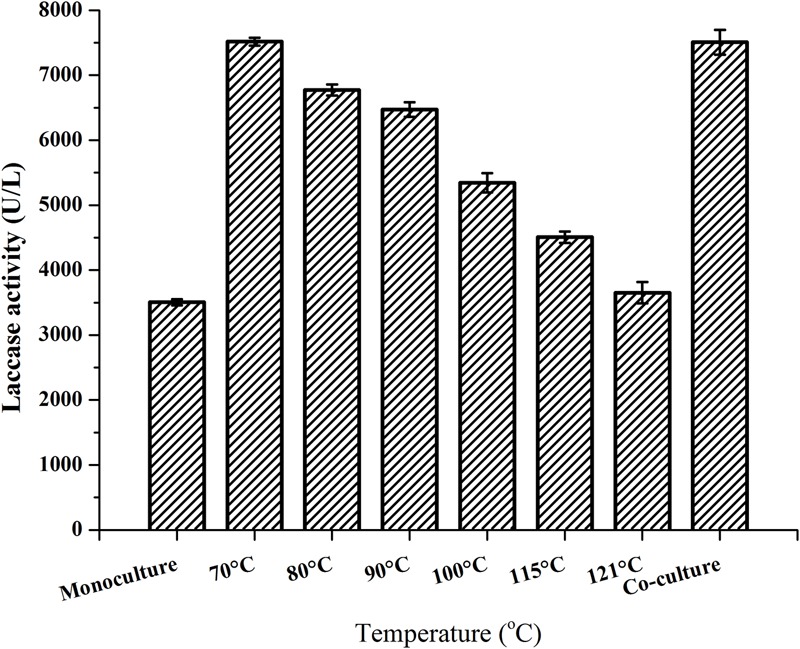
Laccase production by *P. eryngii* var. *ferulae* after adding *R. mucilaginosa* cells exposed to various temperatures.

Our previous study indicated that laccase production by *P. eryngii* var. *ferulae* was enhanced in a co-culture with *R. mucilaginosa* (Wang et al., 2015). The results of that study showed that yeast cells sterilized at 121°C did not improve laccase production. In the present study, we investigated the effect of various sterilization temperatures on the survival percentage of the cells. The results showed that the lowest temperature necessary for the complete sterilization of *R. mucilaginosa* was 70°C (Supplementary Figure 1). Moreover, the reduction of laccase production of *P. eryngii* var. *ferulae* was determined after adding *R. mucilaginosa* cells sterilized at temperatures that varied from 70 to 121°C (**Figure 2**). *R. mucilaginosa* cells sterilized at 121°C showed almost no enhancement of laccase production compared to a *P. eryngii* var. *ferulae* monoculture. These results indicated that the stimulatory compound existed in *R. mucilaginosa* and other yeast cells, and that this compound was temperature-sensitive.

**FIGURE 3:**
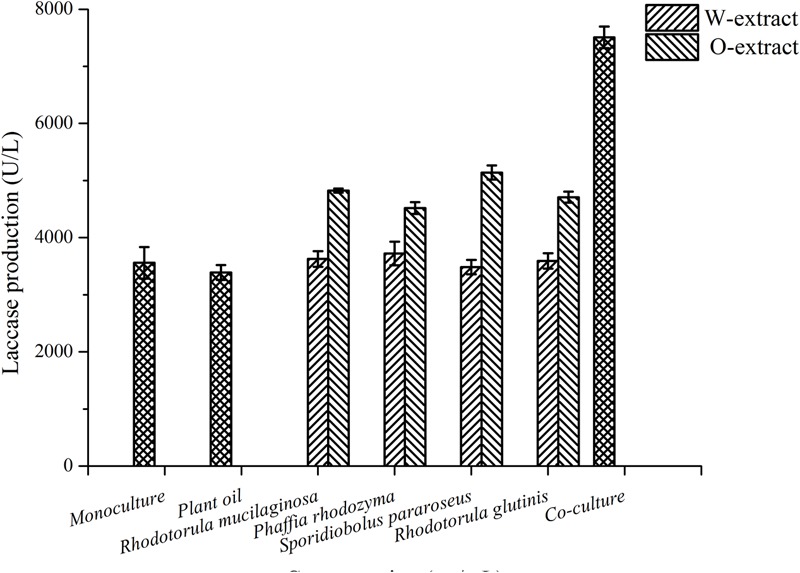
The effect of different extractants of *R. mucilaginosa* on laccase production by *P. eryngii* var. *ferulae*.

To identify the stimulatory compounds, *R. mucilaginosa* cells sterilized at 70°C were treated with various proteases. The results showed that laccase production was not affected by any of the four protease treatments (Supplementary Figure 2). In addition, the sterilized cells were extracted with water and trichloromethane to yield different extracts of *R. mucilaginosa* cells, which were added to a culture containing *P. eryngii* var. *ferulae*. The trichloromethane extracts from *R. mucilaginosa* were dissolved in plant oil (O-extract), and added to the *P. eryngii* var. *ferulae* culture broth. The results showed that the O-extract effectively enhanced laccase production to 5139 U/L by *S. pararoseus* (**Figure 3**). Therefore, the putative compound in the yeast cells was fat-soluble.

### LC-MS Analysis of the Stimulatory Compound in Different Yeasts

**FIGURE 4:**
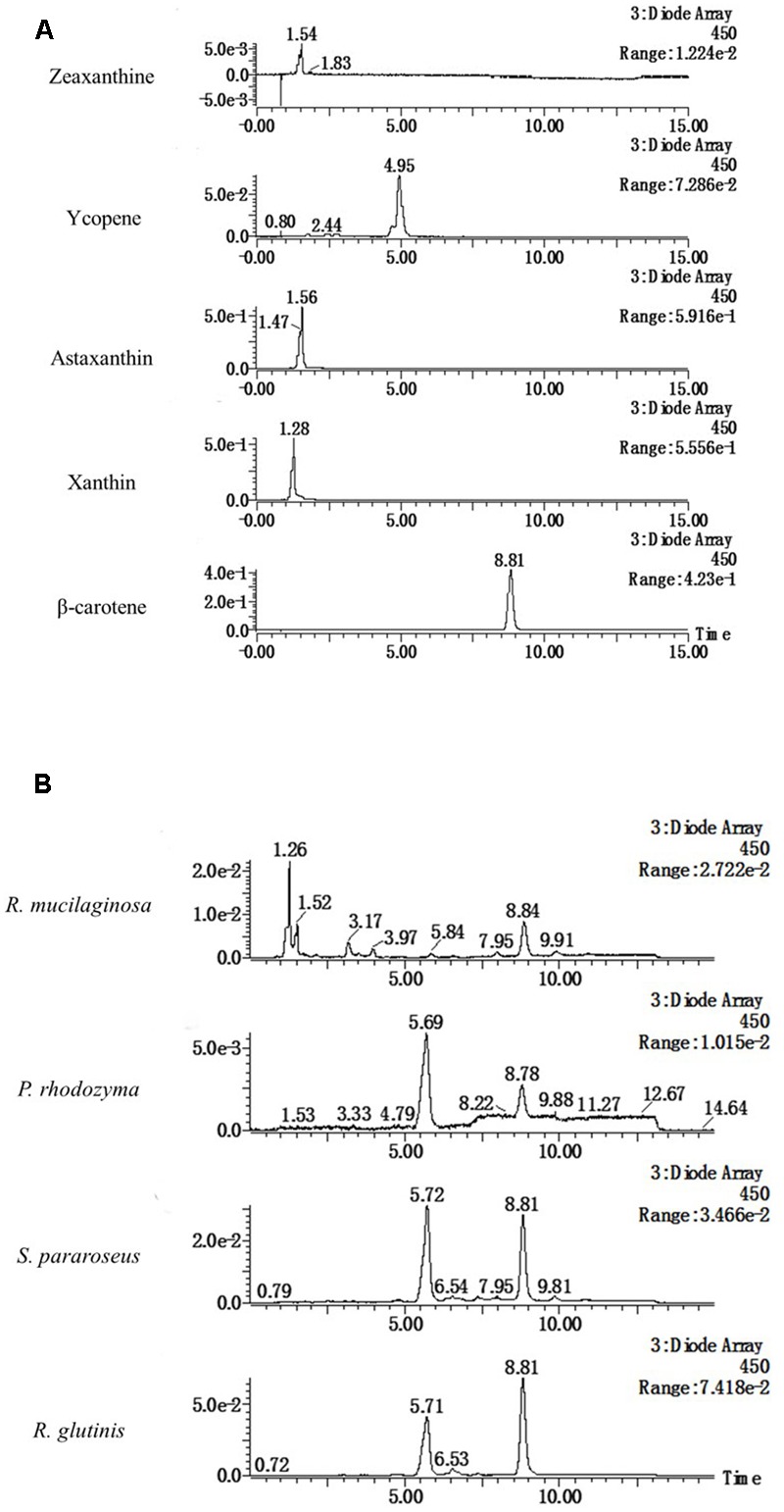
HPLC elution profiles of **(A)** different carotenoid standards and **(B)** the tested yeasts at 450 nm.

**FIGURE 5:**
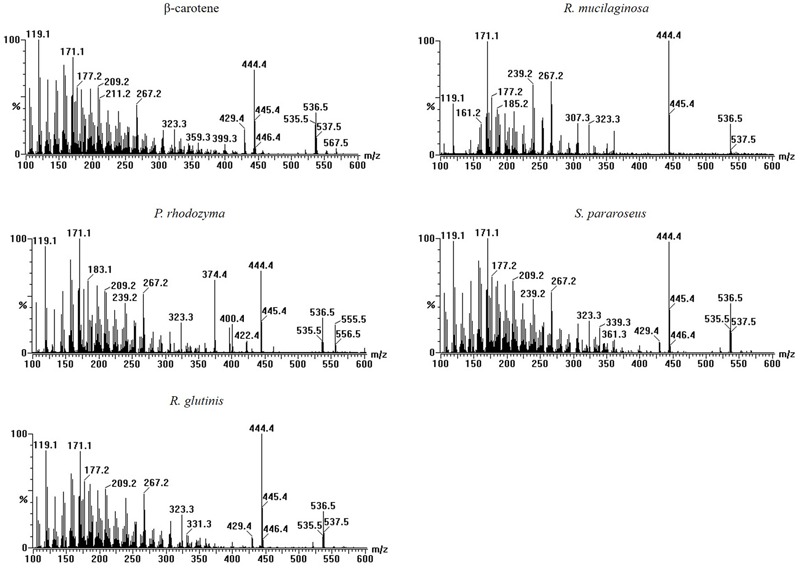
Mass spectra of β-carotene and extracts of four yeasts.

The four yeasts used in this study all enhanced laccase production in *P. eryngii* var. *ferulae* in co-culture. The results from the extracts of the *R. mucilaginosa* cells led us to hypothesize that all of the yeasts used in this study contained the same compounds that enhanced laccase production. To investigate this compound, four yeast cells were treated with liquid nitrogen, and extracted with trichloromethane. The resultant O-extracts from the different yeasts were analyzed by LC-MS. The results showed that all of the different yeast extracts contained the same compound, which had a retention time of 8.8 min (**Figure 4B**). Comparison with standards indicated that this compound had the same retention time as β-carotene (**Figure 4A**). This compound was also identified as β-carotene by its MS spectrum (**Figure 5**).

### Effect of β-Carotene Addition on Laccase Production by *P. eryngii* var. *ferulae*

**FIGURE 6:**
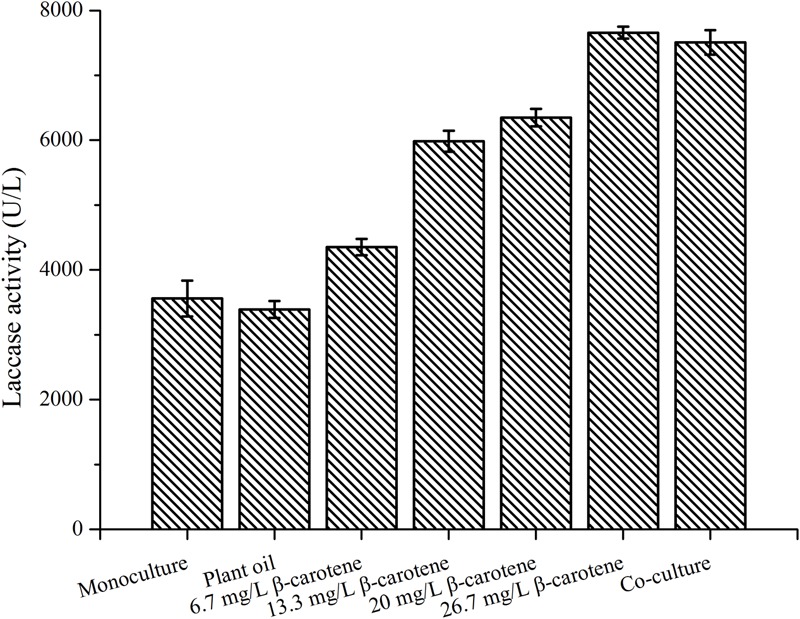
Laccase production by *P. eryngii* var. *ferulae* after adding different concentrations of β-carotene.

To demonstrate that β-carotene was the stimulatory compound in yeast, 1–4 mg of β-carotene dissolved in plant oil was added to 150 mL of *P. eryngii* var. *ferulae* culture broth on day 2. After adding the β-carotene, the laccase production by *P. eryngii* var. *ferulae* at the end of fermentation was increased from 4351 U/L (1 mg β-carotene) to 7656 U/L (4 mg β-carotene), which was higher than the laccase production in a co-culture (**Figure 6**). The highest laccase production after 4 mg β-carotene was added was 2.2-times higher than the control (i.e., a monoculture of *P. eryngii* var. *ferulae*). The laccase production was significantly enhanced by β-carotene, and this result indicated that the stimulatory compound in the four co-cultured yeasts was β-carotene.

### Transcription Level of *lac* in Co-culture

**FIGURE 7:**
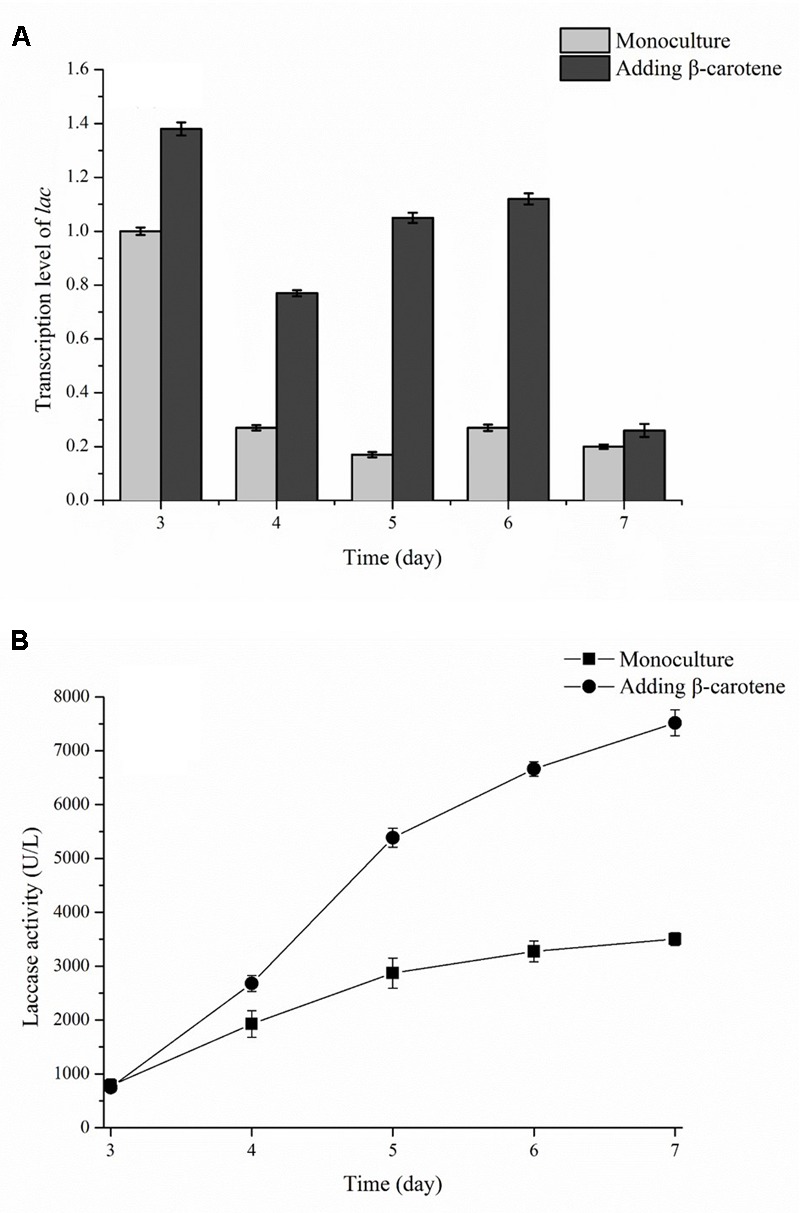
Transcription level of the *lac* gene **(A)** and laccase production **(B)** in a monoculture with or without added β-carotene.

The transcription level of the *lac* gene during submerged fermentation with or without β-carotene was determined and is shown in **Figure 7**. In this experiment, β-carotene was added to the culture broth on day 2, and the laccase activity and the *lac* transcription level were determined on day 3. The results showed that transcription level of *lac* gene peaked on day 3, and then decreased until the end of fermentation. In a monoculture of *P. eryngii* var. *ferulae*, the transcription of *lac* gene decreased sharply and remained at a low level between day 4 and day 7 (**Figure 7A**). In contrast, the *lac* transcription level after β-carotene was added was higher than the control (i.e., a monoculture) from day 3 to 6, and was 6.2-times higher than the control on day 5. Interestingly, the laccase activity of *P. eryngii* var. *ferulae* after β-carotene was added was higher than the control without β-carotene on day 4 (**Figure 7B**), and was 2.1-times higher than the control at the end of culture.

## Discussion

Co-culture has been an effective method to enhance laccase production in several laccase-producing fungi. In Co-culture, laccase-producing strains are always fungi, and other co-cultured strains contained yeasts and other fungi (Wang et al., 2009; Flores et al., 2010; Li et al., 2011). Possible reasons for the enhanced laccase production in a co-culture were investigated, and no clear mechanism for the enhancement was indicated. In our previous study, laccase production by *P. eryngii* var. *ferulae* was enhanced in a co-culture with the yeast *R. mucilaginosa* (Wang et al., 2015). Possible reasons reported in previous studies of other fungi did not provide a convincing explanation for the enhancement of laccase production in a co-culture of *P. eryngii* var. *ferulae* and *R. mucilaginosa*. Therefore, we proposed that the enhancement of laccase production might have been caused by one or several stimulatory compounds that existed in *R. mucilaginosa* cells.

In the present study, three yeasts, *Phaffia rhodozyma, S. pararoseus* and *R. glutinis*, were found to effectively enhance the laccase production by *P. eryngii* var. *ferulae* in a co-culture. An analysis of sterilization temperatures indicated that the stimulatory compound in yeast was temperature-sensitive, which explained why cell extracts from *R. mucilaginosa* sterilized at 121°C had no effect on laccase production (Wang et al., 2015). In addition, *R. mucilaginosa* cells were extracted with water and trichloromethane. The compounds extracted by trichloromethane dissolved in plant oil had a positive effect on laccase production, indicating the stimulatory compound was fat-soluble. All of the yeasts tested in this study similarly improved laccase production, which led us to hypothesize that a common compound that improved laccase production was produced in co-culture. An LC-MS analysis indicated that all four yeasts contained β-carotene, which was identified by comparison with a standard. Moreover, the addition of β-carotene to a *P. eryngii* var. *ferulae* monoculture also improved laccase production, indicating that β-carotene in yeast was the stimulatory compound in the *P. eryngii* var. *ferulae* co-cultures.

β-Carotene is a carotenoid and is commonly used as a coloring agent in the food industry. Some photosynthetic organisms and microorganisms have been reported to be a primary source of carotenoids (Han et al., 2012, 2016; Srinivasan et al., 2015). Some yeasts have excellent carotenoid-producing ability, and efforts have been made to enhance carotenoid production (Shi et al., 2014; Chi et al., 2015). β-Carotene was found as an intermediate in the carotenoid synthesis pathway, and was produced from lycopene (Rodriguez-Saiz et al., 2010; Chi et al., 2015). Yeasts accumulated β-carotene and other carotenoids, allowing the yeasts to be directly used as a food additive. In the present study, β-carotene was contained in the cell extracts of all four yeasts that enhanced laccase production in co-culture, and no other carotenoids were found. The four yeasts were *R. mucilaginosa* (Moline et al., 2012), *Phaffia rhodozyma* (Shi et al., 2014; Chi et al., 2015), *S. pararoseus* (Han et al., 2012, 2016) and *R. glutinis* (Wang et al., 2008; Roadjanakamolson and Suntornsuk, 2010), and have been previously reported as β-carotene-producing strains. Therefore, microbial strains that accumulate β-carotene could be the primary agent that enhances laccase production by fungi in a co-culture.

Laccase is normally found in plants and fungi, and is usually produced by various fungi in submerged fermentation, taking advantage of the ease of manipulation. Various isozymes of laccase have been reported in different fungi in previous studies (Nakatani et al., 2010; Yuan et al., 2016). Hoegger et al. (2006) analyzed the genome of several fungi, and found that some fungi contained different numbers of laccase-encoding genes. These laccase genes were silent or poorly expressed under various culture conditions. The laccase-encoding genes contained a set of promoter regions with various recognition sites for different inducers, including metal ions and aromatic compounds (Piscitelli et al., 2011). β-Carotene is a nutrient and is also used as an antioxidant in food processing. Some oxidants have been used as inducers to enhance laccase production by fungi in submerged fermentation. Such inducers include guaiacol, ferulic acid, butylated hydroxytoluene and phenolic compounds (Kanwal and Reddy, 2011; Wang et al., 2014; Azadfar et al., 2015). Enhancement of fungal laccase production after adding an antioxidant represented a response to oxidative stress, which was caused by oxygen radicals generated by inducers (Thurston, 1994; Wang et al., 2014). A higher concentration of intracellular reactive oxygen species (ROS), including superoxide anion (O_2_^-^), hydrogen peroxide (H_2_O_2_) and hydroxyl radical (OH-), has been reported to enhance both laccase production and transcription, depending on the activation of signal transduction and the oxidative stress (Adekunle et al., 2015; Hu et al., 2016). H_2_O_2_ participated in the activation of the mitogen-activated protein kinase (MAPK) and cyclic adenosine monophosphate (cAMP) pathways (Calvo et al., 2002; Cap et al., 2012). Moreover, the excessive oxidative stress caused by an inducer may lead to the overexpression of laccase, which is a fungal protective response that mitigates oxidative stress (Jaszek et al., 2006). Therefore, the β-carotene in the co-cultured yeasts in this study may enhance laccase production by changing intracellular ROS concentration.

## Author Contributions

CG and ZD designed experiments. CG and LZ performed the experiments. FW, JL, ZD, and GS conceived the project, analyzed data, and wrote the paper.

## Conflict of Interest Statement

The authors declare that the research was conducted in the absence of any commercial or financial relationships that could be construed as a potential conflict of interest.

## References

[B1] Adekunle A. E. Wang F. Hu J. Ma A. Guo C. Zhuang G. (2015). Chitosan multiple addition enhances laccase production from *Trametes versicolor*. *Bioprocess Biosyst. Eng.* 38 1973–1981. 10.1007/s00449-015-1438-z 26178243

[B2] Azadfar M. Gao A. H. Bule M. V. Chen S. (2015). Structural characterization of lignin: a potential source of antioxidants guaiacol and 4-vinylguaiacol. *Int. J. Biol. Macromol.* 75 58–66. 10.1016/j.ijbiomac.2014.12.049 25603142

[B3] Brijwani K. Rigdon A. Vadlani P. V. (2010). Fungal laccases: production, function, and applications in food processing. *Enzyme Res.* 2010:149748 10.4061/2010/149748 PMC2962899 21048859

[B4] Calvo A. M. Wilson R. A. Bok J. W. Keller N. P. (2002). Relationship between secondary metabolism and fungal development. *Microbiol. Mol. Biol. Rev.* 66 447–459. 10.1128/MMBR.66.3.447-459.2002 12208999 PMC120793

[B5] Cap M. Vachova L. Palkova Z. (2012). Reactive oxygen species in the signaling and adaptation of multicellular microbial communities. *Oxid. Med. Cell. Longev.* 2012:976753 10.1155/2012/976753 PMC3395218 22829965

[B6] Chi S. He Y. Ren J. Su Q. Liu X. Chen Z. (2015). Overexpression of a bifunctional enzyme, CrtS, enhances astaxanthin synthesis through two pathways in *Phaffia rhodozyma*. *Microbi. Cell Fact.* 14:90 10.1186/s12934-015-0279-4 PMC4470029 26081576

[B7] Crowe J. D. Olsson S. (2001). Induction of laccase activity in *Rhizoctonia solani* by antagonistic *Pseudomonas fluorescens* strains and a range of chemical treatments. *Appl. Environ. Microbiol.* 67 2088–2094. 10.1128/aem.67.5.2088-2094.2001 11319086 PMC92841

[B8] Dekker R. F. Barbosa A. M. Giese E. C. Godoy S. D. Covizzi L. G. (2007). Influence of nutrients on enhancing laccase production by *Botryosphaeria rhodina* MAMB-05. *Int. Microbiol.* 10 177–185. 10.2436/20.1501.01.25 18075999

[B9] Dong Y. C. Wang W. Hu Z. C. Fu M. L. Chen Q. H. (2012). The synergistic effect on production of lignin-modifying enzymes through submerged co-cultivation of *Phlebia radiata, Dichomitus squalens* and *Ceriporiopsis subvermispora* using agricultural residues. *Bioprocess Biosyst. Eng.* 35 751–760. 10.1007/s00449-011-0655-3 22116528

[B10] Dwivedi P. Vivekanand V. Pareek N. Sharma A. Singh R. P. (2011). Co-cultivation of mutant *Penicillium oxalicum* SAU(E)-3.510 and *Pleurotus ostreatus* for simultaneous biosynthesis of xylanase and laccase under solid-state fermentation. *N. Biotechnol.* 28 616–626. 10.1016/j.nbt.2011.05.006 21642024

[B11] Flores C. Casasanero R. Trejo-Hernandez M. R. Galindo E. Serrano-Carreon L. (2010). Production of laccases by *Pleurotus ostreatus* in submerged fermentation in co-culture with *Trichoderma viride*. *J. Appl. Microbiol.* 108 810–817. 10.1111/j.1365-2672.2009.04493.x 19709340

[B12] Han M. He Q. Zhang W. G. (2012). Carotenoids production in different culture conditions by *Sporidiobolus pararoseus*. *Prep. Biochem. Biotechnol.* 42 293–303. 10.1080/10826068.2011.583974 22708808

[B13] Han M. Xu Z. Y. Du C. Qian H. Zhang W. G. (2016). Effects of nitrogen on the lipid and carotenoid accumulation of oleaginous yeast *Sporidiobolus pararoseus*. *Bioprocess Biosyst. Eng.* 39 1425–1433. 10.1007/s00449-016-1620-y 27145779

[B14] Hoegger P. J. Kilaru S. James T. Y. Thacker J. R. Kues U. (2006). Phylogenetic comparison and classification of laccase and related multicopper oxidase protein sequences. *FEBS J.* 273 2308–2326. 10.1111/j.1742-4658.2006.05247.x 16650005

[B15] Hong Y. Z. Zhou H. M. Tu X. M. Li J. F. Xiao Y. Z. (2007). Cloning of a laccase gene from a novel basidiomycete *Trametes* sp. 420 and its heterologous expression in *Pichia pastoris*. *Curr. Microbiol.* 54 260–265. 10.1007/s00284-006-0068-8 17334840

[B16] Hou H. M. Zhou J. T. Wang J. Du C. H. Yan B. (2004). Enhancement of laccase production by *Pleurotus ostreatus* and its use for the decolorization of anthraquinone dye. *Process Biochem.* 39 1415–1419. 10.1016/s0032-9592(03)00267-x

[B17] Hu J. Wang F. Ma A. Zhuang G. Liu Y. Lu J. (2016). Farnesol stimulates laccase production in *Trametes versicolor*. *Eng. Life Sci.* 16 364–370. 10.1002/elsc.201500082

[B18] Jaszek M. Grzywnowicz K. Malarczyk E. Leonowicz A. (2006). Enhanced extracellular laccase activity as a part of the response system of white rot fungi: *Trametes versicolor* and *Abortiporus biennis* to paraquat-caused oxidative stress conditions. *Pestic. Biochem. Physiol.* 85 147–154. 10.1016/j.pestbp.2006.01.002

[B19] Jeon J. R. Chang Y. S. (2013). Laccase-mediated oxidation of small organics: bifunctional roles for versatile applications. *Trends Biotechnol.* 31 335–341. 10.1016/j.tibtech.2013.04.002 23639526

[B20] Kanwal H. K. Reddy M. S. (2011). Effect of carbon, nitrogen sources and inducers on ligninolytic enzyme production by *Morchella crassipes*. *World J. Microbiol. Biotechnol.* 27 687–691. 10.1007/s11274-010-0507-3

[B21] Klonowska A. Gaudin C. Asso M. Fournel A. Reglier M. Tron T. (2005). LAC3, a new low redox potential laccase from *Trametes* sp. strain C30 obtained as a recombinant protein in yeast. *Enzyme Microb. Technol.* 36 34–41. 10.1016/j.enzmictec.2004.03.022

[B22] Li J. Zhang J. Chen H. Chen X. Lan J. Liu C. (2013). Complete mitochondrial genome of the medicinal mushroom *Ganoderma lucidum*. *PLoS ONE* 8:e72038 10.1371/journal.pone.0072038 PMC3753355 23991034

[B23] Li P. Wang H. L. Liu G. S. Li X. Yao J. M. (2011). The effect of carbon source succession on laccase activity in the co-culture process of *Ganoderma lucidum* and a yeast. *Enzyme Microb. Technol.* 48 1–6. 10.1016/j.enzmictec.2010.07.005 22112763

[B24] Moline M. Libkind D. van Broock M. (2012). Production of torularhodin, torulene, and beta-carotene by *Rhodotorula* yeasts. *Methods Mol. Biol.* 898 275–283. 10.1007/978-1-61779-918-1_19 22711133

[B25] Nakatani M. Hibi M. Minoda M. Ogawa J. Yokozeki K. Shimizu S. (2010). Two laccase isoenzymes and a peroxidase of a commercial laccase-producing basidiomycete, *Trametes* sp. Ha1. *N. Biotechnol.* 27 317–323. 10.1016/j.nbt.2010.02.008 20188874

[B26] Piscitelli A. Giardina P. Lettera V. Pezzella C. Sannia G. Faraco V. (2011). Induction and transcriptional regulation of laccases in fungi. *Curr. Genomics* 12 104–112. 10.2174/138920211795564331 21966248 PMC3129044

[B27] Roadjanakamolson M. Suntornsuk W. (2010). Production of beta-carotene-enriched rice bran using solid-state fermentation of *Rhodotorula glutinis*. *J. Microbiol. Biotechnol.* 20 525–531. 10.4014/jmb.0809.0550 20372023

[B28] Rodriguez-Saiz M. de la Fuente J. L. Barredo J. L. (2010). Xanthophyllomyces dendrorhous for the industrial production of astaxanthin. *Appl. Microbiol. Biotechnol.* 88 645–658. 10.1007/s00253-010-2814-x 20711573

[B29] Shi F. Zhan W. Li Y. Wang X. (2014). Temperature influences beta-carotene production in recombinant *Saccharomyces cerevisiae* expressing carotenogenic genes from *Phaffia rhodozyma*. *World J. Microbiol. Biotechnol.* 30 125–133. 10.1007/s11274-013-1428-8 23861041

[B30] Shraddha Shekher R. Sehgal S. Kamthania M. Kumar A. (2011). Laccase: microbial sources, production, purification, and potential biotechnological applications. *Enzyme Res.* 2011:217861 10.4061/2011/217861 PMC3132468 21755038

[B31] Srinivasan R. Kumar V. A. Kumar D. Ramesh N. Babu S. Gothandam K. M. (2015). Effect of dissolved inorganic carbon on beta-carotene and fatty acid production in *Dunaliella* sp. *Appl. Biochem. Biotechnol.* 175 2895–2906. 10.1007/s12010-014-1461-6 25575588

[B32] Thurston C. F. (1994). The structure and function of fungal laccases. *Microbiology* 140 19–26. 10.1099/13500872-140-1-19

[B33] Wang F. Hu J.-H. Guo C. Liu C.-Z. (2014). Enhanced laccase production by *Trametes versicolor* using corn steep liquor as both nitrogen source and inducer. *Bioresour. Technol.* 166 602–605. 10.1016/j.biortech.2014.05.068 24951276

[B34] Wang H. Peng L. Ding Z. Wu J. Shi G. (2015). Stimulated laccase production of *Pleurotus ferulae* JM301 fungus by *Rhodotorula mucilaginosa* yeast in co-culture. *Process Biochem.* 50 901–905. 10.1016/j.procbio.2015.03.004

[B35] Wang H. L. Yu G. L. Li P. Gu Y. C. Li J. Liu G. S. (2009). Overproduction of *Trametes versicolor* laccase by making glucose starvation using yeast. *Enzyme Microb. Technol.* 45 146–149. 10.1016/j.enzmictec.2009.04.003

[B36] Wang S. L. Chen D. J. Deng B. W. Wu X. Z. (2008). Effects of high hydrostatic pressure on the growth and beta-carotene production of *Rhodotorula glutinis*. *Yeast* 25 251–257. 10.1002/yea.1583 18338316

[B37] Yuan X. Tian G. Zhao Y. Zhao L. Wang H. Ng T. B. (2016). Biochemical characteristics of three laccase isoforms from the basidiomycete *Pleurotus nebrodensis*. *Molecules* 21:E203 10.3390/molecules21020203 PMC6273344 26861278

