# Supplementary material for: β-Carotene from Yeasts Enhances Laccase Production of Pleurotus eryngii var. ferulae in Co-culture
Source: Front Microbiol. 2017 Jun 16;8:1101. doi: 10.3389/fmicb.2017.01101 (PMC5472667; doi:10.3389/fmicb.2017.01101)
Supplement: Supplementary file 1 [file Table_1.PDF]

β-Carotene from yeasts enhances laccase production of *Pleurotus eryngii* var. *ferulae* in co-culture

Supplementary materials

Table 1 Gradient program used in the chromatographic separation of yeast extracts.

Time (min)	A (%, v/v)	B (%, v/v)
Initial	0.0	100.0
5.0	0.0	100.0
10.0	10.0	90.0
12.0	10.0	90.0
12.1	0.0	100.0
15.0	0.0	100.0
