# Supplementary material for: β-Carotene from Yeasts Enhances Laccase Production of Pleurotus eryngii var. ferulae in Co-culture
Source: Front Microbiol. 2017 Jun 16;8:1101. doi: 10.3389/fmicb.2017.01101 (PMC5472667; doi:10.3389/fmicb.2017.01101)
Supplement: Supplementary file 2 [file Table_2.PDF]

β-Carotene from yeasts enhances laccase production of *Pleurotus eryngii* var. *ferulae* in co-culture

Supplementary materials

Table 2. Primer sequences for cDNA amplification.

mRNA	Primers
lac1	Forward: GCCTTCCTTCGTTAACCAGT<br>Reverse: ACAAGTGAGAGTGGTACCAG
β-actin	Forward: GTCTGGATTGGTGGTTCTAT<br>Reverse: CCTGACTCGTCGTATTCTTG
