# Supplementary material for: β-Carotene from Yeasts Enhances Laccase Production of Pleurotus eryngii var. ferulae in Co-culture
Source: Front Microbiol. 2017 Jun 16;8:1101. doi: 10.3389/fmicb.2017.01101 (PMC5472667; doi:10.3389/fmicb.2017.01101)

β-Carotene from yeasts enhances laccase production of *Pleurotus eryngii* var. *ferulae* in co-culture

Supplementary materials

Figure 1 *R. mucilaginosa* sterilized by various temperatures. 1: untreated, 2: 55 ℃, 3: 60 ℃, 4: 65 ℃, 5: 70 ℃.

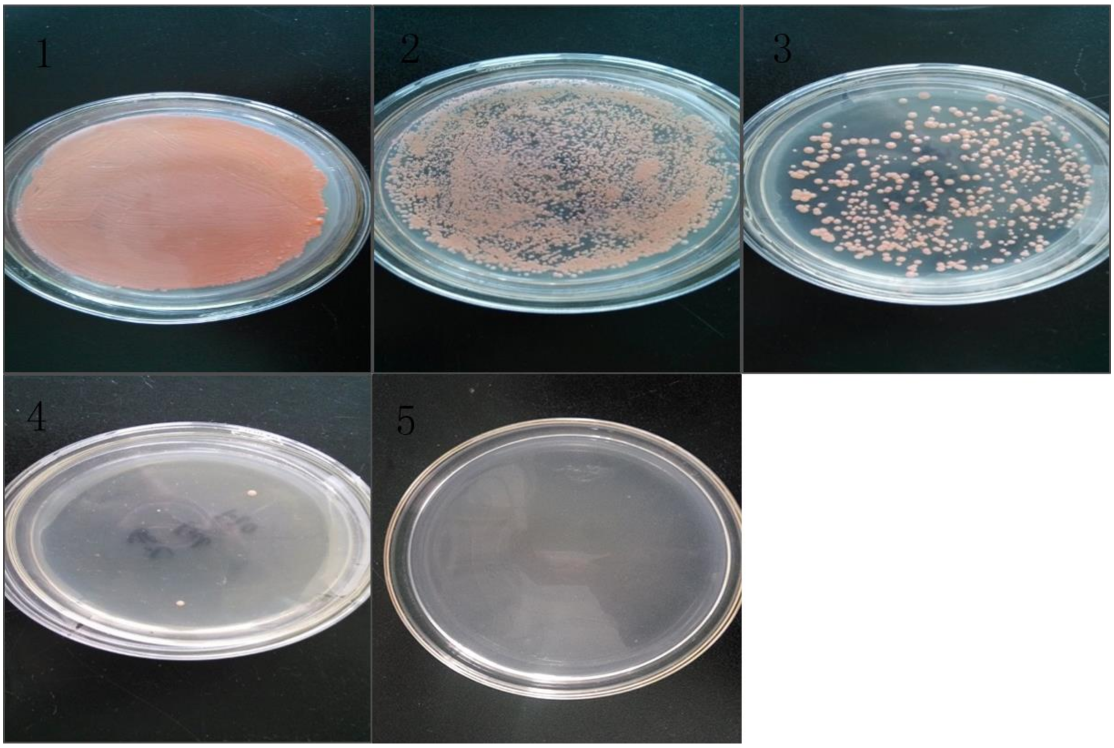

Supplement: Supplementary file 3 [file Image_1.PDF]
