# Supplementary material for: β-Carotene from Yeasts Enhances Laccase Production of Pleurotus eryngii var. ferulae in Co-culture
Source: Front Microbiol. 2017 Jun 16;8:1101. doi: 10.3389/fmicb.2017.01101 (PMC5472667; doi:10.3389/fmicb.2017.01101)

β-Carotene from yeasts enhances laccase production of *Pleurotus eryngii* var. *ferulae* in co-culture

Supplementary materials

Figure 2 Laccase production after adding 4 g various protease treated *R. mucilaginosa* cells.

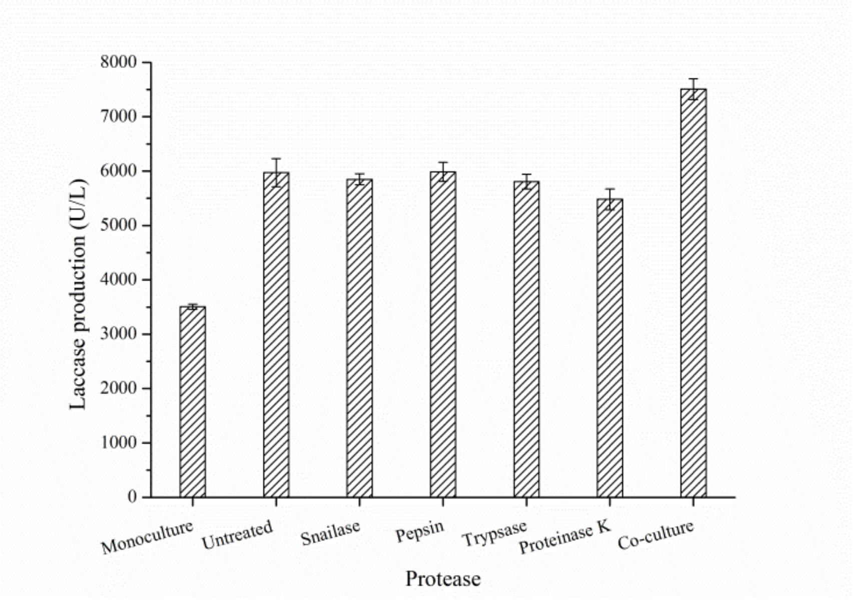

Supplement: Supplementary file 4 [file Image_2.PDF]
